# 

**Published:** 1880-03

**Authors:** 


### CONTENTS FOR MARCH, 1880.

ORIGINAL COMMUNICATIONS.

ART. I.—Mysophobia. By WILLIAM A. HAMMOND, M. D. .......... 115
ART. II.—Water Conductors and the Artesian Well at Charleston, S. C. By MIDDLETON MICHEL, M. D. .......... 126
ART. III.—Deafness the Result of Sympathy between the Ear, Teeth and Mouth. By LAURENCE TURNBULL, M. D. .......... 133
ART. IV.—The Audiphone. By J. ALLEN OSMUN, M. D. .......... 135
ART. V.—The Use of Hydrobromic Ether in Labor. By LAURENCE TURNBULL, M. D. .......... 136

TRANSLATIONS.

ART. I.—Spermatic Hydrocele. Translated from the German by JAMES AUMANN, M. D. .......... 137
ART. II.—Physiological Action of Sulphate of Quinine. Translated from the French by BOYLAND .......... 140

ECLECTIC DEPARTMENT.

Abuse of Medical Charities, and Unprofessional Conduct of Clinical Examiners .......... 142
How to Succeed in Our Profession .......... 145
Gold Fillings Sixty-Four Years Old .......... 147

EDITORIAL DEPARTMENT.

Hospitals, their Institution, Management, etc. .......... 148
Medical Fees, etc. Free Dispensaries .......... 150
Papers Read before Medical and Dental Societies .......... 151
University of Maryland, etc. .......... 152
University of Pennsylvania .......... 157
To Our Dental Friends .......... 154

BIBLIOGRAPHICAL DEPARTMENT .......... 154

EXCERPTA.

Doctor vs. Apothecary. A Crusade against Quackery .......... 158
Doctors in Memphis during Yellow Fever .......... 159
Jaborandi in Dropsy. Quebracho in Dyspnœa .......... 159
Treatment of Chronic Nervous Exhaustion .......... 159
Anæmic Menorrhagia .......... 160
Attenuations. Collecting Bills. Pruritus Ani .......... 161
Sweating Hands or Feet. Hymeneal. Natalitial. Necrological .......... 162
Report of Deaths and Interments in Baltimore for February .......... 163
Meteorological Summary for February .......... 165
Iodide of Starch in Poisoning .......... 165
Of Individual Importance to Every One Interested in Dentistry .......... 166
Domestication and Brain Growth .......... 170

